# Effect of *Morus alba* L. Fruit Extract on Sperm Quality, Testosterone Profile, and Testicular Histology in Male Rats

**DOI:** 10.3390/life16060991

**Published:** 2026-06-12

**Authors:** Tarinee Sawatpanich, Sararat Innoi, Arada Chaiyamoon, Supatcharee Arun, Nareelak Tangsrisakda, Chadaporn Chaimontri, Therachon Kamollerd, Sineenad Teerapatpaisan, Natsajee Nualkaew, Alexander T. H. Wu, Sitthichai Iamsaard

**Affiliations:** 1 Department of Anatomy, Faculty of Medicine, Khon Kaen University, Khon Kaen 40002, Thailand; tarinee@kku.ac.th (T.S.); sararat.i@kkumail.com (S.I.); aradch@kku.ac.th (A.C.); supatar@kku.ac.th (S.A.); nareelak@kku.ac.th (N.T.); chadaporn_chaimontri@kkumail.com (C.C.); therachon_k@kkumail.com (T.K.); 2 Faculty of Pharmaceutical Sciences, Khon Kaen University, Khon Kaen 40002, Thailand; sineenad.te@kkumail.com (S.T.); nnatsa@kku.ac.th (N.N.); 3 International Ph.D. Program for Translational Science, College of Medical Science and Technology, Taipei Medical University, Taipei 11031, Taiwan; chaw1211@tmu.edu.tw; 4 The Ph.D. Program of Translational Medicine, College of Medical Science and Technology, Taipei Medical University, Taipei 11031, Taiwan; 5 Clinical Research Center, Taipei Medical University Hospital, Taipei Medical University, Taipei 11031, Taiwan; 6 Graduate Institute of Medical Sciences, National Defense Medical University, Taipei 11490, Taiwan; 7 Research Institute for Human High Performance and Health Promotion, Khon Kaen University, Khon Kaen 40002, Thailand

**Keywords:** *Morus alba*, cyanidin-3-glucoside, antioxidant, reproductive toxicity, acrosome reaction

## Abstract

*Morus alba* L. fruit extract (MFE), which is rich in cyanidin 3-glucoside (C3G), demonstrates antioxidant properties and pharmacological effects, but its reproductive safety remains poorly understood. Polyphenols modulate steroidogenesis, spermatogenesis, and sperm acrosome integrity; therefore, toxicity assessment is needed for use. This study aimed to evaluate the antioxidant profiles and subchronic reproductive effects of MFE. MFE was standardized using high-performance liquid chromatography (HPLC), 2,2-diphenyl-1-picrylhydrazyl (DPPH), and ferric reducing antioxidant power (FRAP) assays. Male rats were administered MFE (250 or 500 mg/kg BW) for 56 days. Assessments included computer-assisted sperm analysis (CASA), testosterone, seminal fructosamine, and testicular CYP11A1 and androgen receptor (AR) expression. Acrosomal status was determined using PNA lectin staining. The results showed that MFE contained C3G (119.42 mg/g), antioxidant capacity (DPPH IC_50_: 0.101 mg/mL; FRAP: 465.01 µmol Fe (II)/g), and total contents (phenolics: 41.15 mg GAE/g; flavonoids: 3.15 mg CE/g; anthocyanidins: 11.04 mg C3G/g). MFE did not alter testicular histology and seminiferous stages VII-IX. High doses significantly increased sperm concentration, while both doses reduced sperm beat cross frequency. Testosterone, fructosamine, and CYP11A1/AR expressions showed increasing trends. Significantly, high doses induced a precocious acrosome reaction. In conclusion, MFE has no reproductive toxicity and pro-fertility effects on sperm quantity or androgenic markers, supporting safe subchronic use.

## 1. Introduction

*Morus alba* L. (white mulberry) has been widely cultivated in many tropical countries including Thailand, primarily for traditional medicinal use. Its ripe fruit has been documented to be remarkably rich in polyphenolic compounds, flavonoids, and anthocyanins [1,2]. In particular, cyanidin 3-glucoside, a major anthocyanin, has been identified as the predominant bioactive constituent in ripe mulberry fruit extract (MFE), exhibiting potent antioxidant activity [3]. These biological compounds included total phenolic and total flavonoid contents [3,4]. The pharmacological effects of MFE have been demonstrated to include anti-atherosclerotic, antidiabetic, antihyperglycemic, anti-obesity, neuroprotective, and hepatoprotective effects [5,6]. However, anthocyanin has been demonstrated to bind estrogen receptor alpha and beta, as well as exert its estrogenic activity in vitro [7,8]. In addition, the exogenous estradiol can inhibit steroidogenesis via P450c17 [9,10]. Moreover, previous studies revealed the effects of phytoestrogens on the male reproductive system by decreasing blood testosterone levels, sperm concentration, and the percentage of normal sperm, via the activation of estrogen receptors [11,12,13,14,15]. Therefore, plant-derived estrogenic flavonoids can interact with male steroid hormone biosynthesis, receptor signaling, and gametogenic processes. However, the potential reproductive toxicity of Thai MFE has never been investigated [16,17].

Oxidative stress within the male reproductive system is recognized as a major etiological factor contributing to male infertility, as it adversely affects spermatogenesis, induces sperm DNA fragmentation, impairs sperm motility, and compromises acrosomal integrity [18]. Several studies have demonstrated that anthocyanins and polyphenols derived from MFE and leaves can modulate gonadal oxidative stress and male steroidogenesis [18,19]. Furthermore, anthocyanins such as cyanidin 3-glucoside can protect testicular function under oxidative stress conditions [20]. Indeed, polyphenol-rich plant extracts can ameliorate testicular damage induced by chemotoxic agents [21,22]. However, the effects of MFE on the intact male reproductive system following subchronic exposure have not yet been comprehensively investigated. It has been reported that, owing to their structural similarity to phytoestrogens, polyphenolic compounds may exert dose-dependent effects on testosterone synthesis and spermatogenesis through the seminiferous epithelium cycle [23,24]. Moreover, the sperm acrosome represents a sensitive indicator of sperm maturation integrity, and its precocious acrosome reaction (AR) within the cauda epididymis has been linked to exposure to phytochemical compounds or plant extracts [25,26]. It is known that testosterone synthesized via CYP11A1-catalyzed conversion is essential for spermatogenesis, accessory gland function, and libido. These physiological effects are primarily mediated through activation of the androgen receptor [27,28,29]. Spermiogenesis, which involves nuclear condensation, acrosome biogenesis, and axoneme assembly, occurs predominantly during stages VII–IX in seminiferous tubules in rats [30]. Furthermore, seminal vesicle fructosamine represents an important biochemical marker of androgenic responsiveness and is positively associated with elevated testosterone levels [31].

The reproductive toxicity and steroid-sensitive bioactivity of the principal constituent of Thai MFE have not been fully reported, despite escalating consumer use. This study, therefore, aimed to provide a reproductive toxicity assessment of Thai MFE in adult male rats in order to contribute to an evidence-based safety evaluation.

## 2. Materials and Methods

### 2.1. Plant Material and Extract Preparation

Ripe mulberry fruits (MF) were purchased form Baan Suan Piemsuk farm, Ubolratana District, Khon Kaen Province, Thailand (16°49′20.8″ N 102°38′06.1″ E). The 1000 g of MF was mixed with 1000 mL of distilled water (DW) and blended using an electric blender. Then, the homogenate mixture was heated at 60 °C for 30 min, cooled down, filtered, and stored at −20 °C. The filtered mulberry fruit extract (MFE) was lyophilized using a freeze-dryer at the Kasetsart Agricultural and Agro-Industrial Product Improvement Institute (KAPI). The MFE powder extract was calculated as a percentage yield of 5.178% based on the following formula: % yield = (Weight of MFE powder (g))/(Weight of mulberry fruit (g)× 100 [32].

### 2.2. Identification of Cyanidin 3-glucoside in MFE Using HPLC

High-performance liquid chromatography (HPLC) analysis was performed using an autosampler system (Agilent 1260, Agilent Technologies, Santa Clara, CA, USA). Peaks were monitored using a UV diode array detector at a wavelength of 280 nm. Chromatographic separation was achieved on a reversed-phase RP-18 column (Synergi™ 4 µm Fusion-RP 80 Å, 250 × 4.6 mm, 5 µm; Phenomenex, Torrance, CA, USA). The FME sample solution (2.5 mg/mL) was injected at a volume of 10 µL. The mobile phase consisted of solvent A (0.2% formic acid in deionized water) and solvent B (0.2% formic acid in acetonitrile), delivered at a flow rate of 0.8 mL/min. The gradient program was applied as follows: the initial composition was 100% A at 0 min, which was linearly changed to 85% A and 15% B at 5 min, then to 50% A and 50% B at 10 min, followed by 20% A and 80% B at 15 min. The composition was further adjusted to 100% B at 20 min and maintained until 25 min, after which the system was returned to the initial conditions (100% A) at 30 min for column re-equilibration. The total run time was 30 min.

### 2.3. Phytochemical Analysis

#### 2.3.1. Total Phenolic Content Determination

Total phenolic content was determined using the Folin–Colorimetric method, adapted from [33]. Briefly, MFE was added to DW and Folin–Ciocalteu reagent. After 6 min of incubation, 7% sodium carbonate and DW were added and incubated for 90 min. Absorbance was read at 760 nm using a spectrophotometer in triplicate. Total phenolic content was tabulated form a gallic acid calibration curve (15–275 µg/mL) and reported as milligrams of gallic acid equivalents per gram of MFE (mg GAE/g sample).

#### 2.3.2. Total Flavonoid Content Determination

Total flavonoid content (TFC) was determined as previously described [33]. Briefly, the MFE sample was mixed with DW and 5% sodium nitrite for 5 min. Then, 10% aluminum chloride was added, and the reaction mixture was incubated for 6 min. After that, 1M sodium hydroxide and DW were added and absorbance was read at 510 nm using a spectrophotometer in triplicate. TFC was calculated from the catechin calibration curve (15–300 µg/mL) and expressed as milligrams of catechin equivalents per gram of sample (mg CE/g sample).

#### 2.3.3. Total Anthocyanidin Content Determination

In brief, 0.025 M potassium chloride buffer (pH 1.0) and 0.4 M sodium acetate buffer (pH 4.5) were prepared and adjusted to a final volume of 1 L using DW. Total anthocyanins were measured and expressed as cyanidin-3-glucoside [34]. Briefly, MFE sample aliquots were transferred to volumetric flacks and diluted to 25 mL with the pH 1.0 buffer and the pH 4.5 buffer. After that, the solutions were kept in the dark for 15 min and measured for absorbance using a spectrophotometer at 510 and 700 nm. The measurements were performed in triplicate. Finally, the absorbance values were used to calculate anthocyanin content (mg/g).

### 2.4. Antioxidant Activity Assays

#### 2.4.1. DPPH Radical Scavenging Assay

Antioxidant activity was evaluated using the 2,2-diphenyl-1-picrylhydrazyl (DPPH) radical scavenging assay [35]. Sample solutions were prepared at five concentrations (0.03, 0.06, 0.09, 0.12, and 0.15 mg/mL) and mixed with a 0.1 mM DPPH solution in 95% ethanol. The mixture solutions were incubated in the dark at room temperature for 30 min and absorbance was read at a 517 nm using a spectrophotometer (in triplicate). Butylated hydroxytoluene (BHT), α-tocopherol, and ascorbic acid were used as standard controls. The absorbance value of the MFE sample was used to calculate the percentage (%) of DPPH radical scavenging activity. After that, the percentage of radical scavenging activity for each sample concentration was plotted to determine the half-maximal inhibitory concentration (IC_50_).

#### 2.4.2. Ferric Reducing Antioxidant Power (FRAP) Assay

The antioxidant activity of MFE was determined using the ferric reducing antioxidant power (FRAP) assay [36]. The FRAP reagent was prepared by mixing 300 mM of acetate buffer with 20 µM FeCl_3_·6H_2_O and 2,4,6-tris(2-pyridyl)-s-triazine (TPTZ) dissolved in 40 µM HCl and DW. The mixture solution was incubated at 37 °C for 4 min; then, we measured its absorbance at 593 nm using a spectrophotometer (in triplicate). Butylated hydroxytoluene (BHT), α-tocopherol, and ascorbic acid were used as standards. Antioxidant capacity was calculated from the MFE sample absorbance using a calibration curve of ferrous sulfate (FeSO_4_·7H_2_O) over 0.1–1 mM and reported as µmol Fe (II) per g of sample.

### 2.5. Experimental Animals and Design

Adult male Wistar rats (N = 24; body weight: 270–350 g) were procured from Nomura Siam International Co., Ltd. (Bangkok, Thailand). They were housed in an animal shoebox (26.6 × 42.5 × 18.5 cm) and fed with a commercial pelleted diet throughout the experimental periods. Rats were maintained at 23 ± 2 °C and under a 12 h light/dark cycle. This study was approved for animal ethics by the Institutional Animal Care and Use Committee, Northeast Laboratory Animal Center, Khon Kaen University, (Code: IACUC-KKU-92/67). Animals were randomly divided into three groups (Control, MFE250, and MFE500), with each group consisting of 8 animals. During the treatment period, the MFE groups orally received MFE at 250 or 500 mg/kgBW and the control rat received DW for 56 consecutive days (one cycle of rat spermatogenesis). Body weights were recorded daily during the experimental period, and final body weights were measured before euthanasia.

### 2.6. Sample Collections

Rats were anesthetized (thiopental sodium at 40 mg/kg, i.p.) and euthanized via cervical dislocation in accordance with established ethical standards and animal welfare guidelines. Then, the testes, epididymis, vas deferens, prostate gland, and seminal vesicle were collected and weighed to record them as absolute weight. For relative weight analysis, each mass of reproductive organs was calculated in relation to the total body weight (g/100 gBW).

### 2.7. Blood Collection and Serum Testosterone Determination

After the rats had been anesthetized, the abdominal and thoracic walls were opened for blood collection via cardiac puncture at the left ventricle of the heart. The blood samples were centrifuged at 12,000 rpm for 10 min at 4 °C, after which the serum was rapidly separated. The serum testosterone concentration was then determined via electrochemiluminescence immunoassays at the clinical laboratory section, Srinagarind Hospital, Khon Kaen University.

### 2.8. Sperm Quality Analysis

The epididymal sperm was collected from the left caudal epididymis and diluted in 2 mL of Krebs–Ringer solution (Themo Fischer Scientific Inc., Waltham, MA , USA) before incubation for 30 s at room temperature [21]. After that, the sperm qualities, including motility, progressive motility, concentration, amplitude of lateral head displacement (ALH), beat cross frequency (BFC), linearity (LIN), straightness (STR), average path velocity (VAP), curvilinear velocity (VCL), and straight line velocity (VSL), were analyzed using computer-assisted sperm analysis (CASA IVOS^®^ II, Hamilton Thorne Inc., Beverly, MA, USA).

### 2.9. Seminal Vesicle Fluid (SVF) Collection and Fructosamine Level Analysis

The left seminal vesicle was collected and cut at the mid-transverse plane to obtain the SVF using a previously reported method [37]. The SVF secretion percentage was calculated as previously described [38] as follows: SVF secretion percentage (%) = (SVF [g])/(total seminal vesicle gland [g]) × 100. Subsequently, the SVF was diluted with 0.9% saline solution and the fructosamine level was determined using electrochemiluminescence immunoassays (Roche Cobas^®^ e801 Immunoassay, Basel, Switzerland) at the clinical laboratory section, Srinagarind Hospital, Khon Kaen University.

### 2.10. Histological Examination

After being fixed with 10% formalin for 48 h, the right testis was routinely processed for sectioning. Briefly, tissue sections were air-dried on a hot plate at 60 °C for 10 min to ensure adherence of tissue and removal of residual moisture. Paraffins were removed by incubating the slides in two changes of xylene (Xylene I and Xylene II) followed by rehydration through a graded ethanol series (100%, 95%, and 70%) and, finally, washing in DW for 5 min each. Hematoxylin and eosin (H&E) staining was performed. Then, stained sections were used to evaluate the testicular histology between treated and untreated groups. For each section, five different fields were selected using the Zen 2 lite program (ZEISS, Oberkochen, Germany) and images were captured with an AxioCam ICc 5 digital camera (ZEISS, Oberkochen, Germany) under a 20× objective lens.

### 2.11. Analysis of Seminiferous Tubule Stages VII–IX Using Peanut Agglutinin (PNA) Lectin Staining

The testicular sections were evaluated for tubular stages via PNA lectin staining. Briefly, tissue slides were incubated in 1× Phosphate buffered saline (PBS) buffer and subjected to microwave heating (560 W) for 3 times of 5 min each to retrieve antigens. The slides were cooled down at room temperature for 20 min and washed three times with 1× PBS (3 min per wash). Non-specific binding was blocked by incubating the slides with 1% BSA in PBS for 30 min at room temperature, followed by three additional washes in 1× PBS. The slides were then incubated with PNA lectin with Alexa Fluor™ 488-conjugated Peanut Agglutinin (1:600, PNA, Cat. No. L21409, Thermo Fisher Scientific Inc., Waltham, MA, USA) for 45 min in dark room and subsequently washed three times with 1× PBS (3 min each). Then, the slides were mounted with VECTASHIELD^®^ containing DAPI (4′,6-diamidino-2-phenylindole, Cat. No. H-1200-10, Vector Laboratories, Newark, CA, USA). Finally, two hundred seminiferous tubules were observed under a Nikon Eclipse Ni LED fluorescent microscope (Nikon, Tokyo, Japan). For analysis, seminiferous tubules stages VII–IX (the most critical transition phases, representing spermatogenesis acceleration) were counted and expressed as percentage [39].

### 2.12. Analysis of Acrosome Status of Sperm Using Peanut Agglutinin (PNA) Lectin Staining

The acrosome was visualized via Peanut Agglutinin (PNA) lectin staining to distinguish intact and reacted sperm, as previously described [40]. The diluted sperm in PBS (1:1, 20 μL) was incubated in dark eppendorf tubes containing 100 μL of Alexa Fluor™ 488-conjugated Peanut Agglutinin (PNA, 1:600, Cat. No. L21409, Thermo Fisher Scientific Inc., Waltham, MA, USA) at 37 °C for 45 min to visualize positive immunoreactivity (green fluorescence). Then, mixed sperm was smeared on glass slides and dried in a dark room. After that, the smeared sperm was washed in PBS for 3 min and counterstained with Hoecsth 33342 (Abcam, Cambridge, UK) to visualize the nuclei (blue fluorescence). Finally, the sperm was washed in PBS for 3 min and mounted with glycerol. The lectin selectively binds to terminal β-galactose residues on the outer acrosomal membrane. Two hundred sperm were observed and counted using a Nikon Eclipse Ni LED fluorescence microscope (Nikon, Tokyo, Japan). Acrosome-intact sperm exhibited strong, intense green fluorescence across the acrosomal region, whereas acrosome-reacted sperm showed weak or absent staining. The percentage of acrosome-reacted and intact sperm was calculated as previously described [41].

### 2.13. Western Blotting Analysis

The decapsulated testis was mixed with lysis buffer (1× radioimmunoprecipitation assay [RIPA] solution [Cell Signaling Technology, Danvers, MA, USA] containing protease inhibitor cocktails [Sigma-Aldrich, Burlington, MA, USA]). The testicular mixture was homogenized and centrifuged at 4 °C and 12,000 rpm for 10 min before collecting the supernatant. Total protein concentration (ug/ul) in the supernatant was determined at a wavelength of 280 nm using the NANO drop spectrophotometer (ND-100, Nanodrop technologies, Wilmington, DE, USA).

Total extracted testicular proteins (120 µg/lane) were separated and transferred onto nitrocellulose membrane (0.2 μm pore size). After that, protein membranes were incubated with 5% bovine serum albumin (BSA) to block non-specific binding proteins. All membranes were incubated (4 °C, overnight) with mouse anti-AR (1:1000 dilution; Santa Cruz Biotechnology, Inc., Dallas, TX, USA, Cat. No. sc-7305), goat anti-CYP11A1 (1:1000 dilution; Santa Cruz Biotechnology, Inc., Dallas, TX, USA; Cat. No. AP160P) antibodies before incubation with their specific secondary antibodies, including Anti-Mouse HRP conjugate (1:10,000 dilution; Merck, Rahway, NJ, USA; Cat. No. sc-18040) at room temperature for 1 h and the GAPDH (1:1000 dilution; Abcam, Cambridge, UK; Cat No. ab8245), was used as an internal control. The immunoreactive bands were detected using a enhance chemiluminescence (ECL) substrate reagent kit (GE Healthcare Life Science, C—Chicago, IL, USA) and captured under Gel Documentation 4 (ImageQuant 600, GE HealthCare, Chicago, IL, USA). Finally, the ImageJ program (Version 1.5i, National Institute of Health, Bethesda, MD, USA) was used to quantify protein band intensities.

### 2.14. Statistical Analysis

All data are expressed as mean ± standard deviation (SD), using the GraphPad Prism 8 (GraphPad Software, Boston, MA, USA) for analysis. The ANOVA and Tukey’s post hoc tests were used to compare the difference between groups and a *p*-value less than 0.05 was considered for statistical significance.

## 3. Results

### 3.1. Identification of Active Compounds Using HPLC

**Figure 1:**
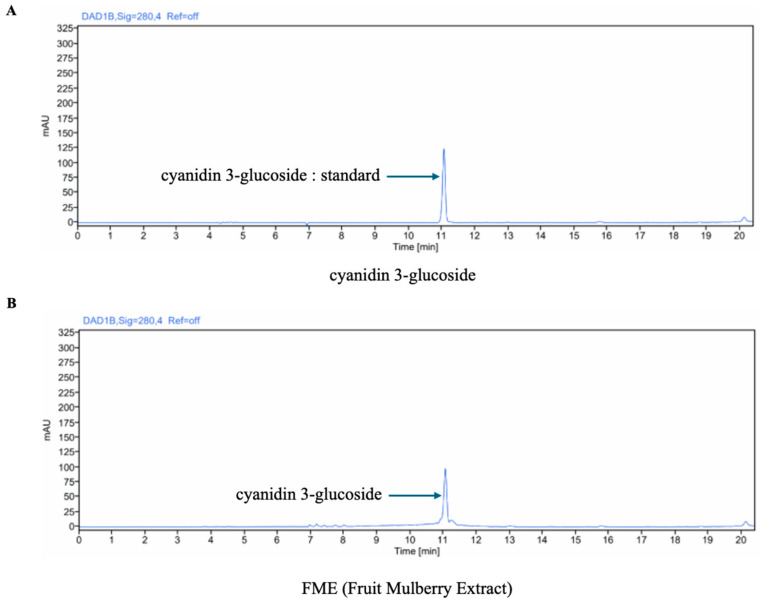
The HPLC peak of cyanidin 3-glucoside determined in a standard control (**A**) and found in Thai MFE (**B**).

The results show that the aqueous MFE used in this study contains a specific peak of cyanidin 3-glucoside, as determined by HPLC (Figure 1). In addition, the concentration of cyanidin 3-glucoside found in the Thai MFE was quantified at approximately 119.42 ± 0.24 mg/g MFE.

### 3.2. Antioxidant Capacity in MFE

As compared to the antioxidant standard controls, Table 1 shows that Thai MFE has antioxidant capacities, as determined using DPPH (IC_50_, 0.101 ± 0.002 mg/mL) and FRAP assays (465.01 ± 8.04 µmol of Fe (II)/g sample). According to the colorimetric analyses, the total phenolic, flavonoid, and anthocyanidin contents found in MFE are approximately 41.15 ± 0.71 mg GAE/g, 3.15 ± 0.11 mg CE/g, and 11.04 ± 0.01 mg cyanidin-3-glucoside/g, respectively.

### 3.3. Effect of MFE on Reproductive Organ Weights and Sperm Qualities

After administration with MFE for 56 consecutive days, both doses of MFE did not change the final body weight and weights of the testis, seminal vesicle, and prostate gland of adult male rats as compared to those of the control (Table 2). Significantly, MFE increased the relative epididymal weight in the MFE500 group compared to the control. For the sperm parameters, the total and progressive sperm motilities did not differ among groups. However, MFE (500 mg/ kg) significantly increased the sperm concentration (38.71 ± 15.04 million/mL) compared to the control group (Table 3). Moreover, the beat cross frequency (BFC) of rat sperm determined using CASA was significantly reduced in both MFE-treated groups as compared to the control (Table 3).

**Figure 2:**
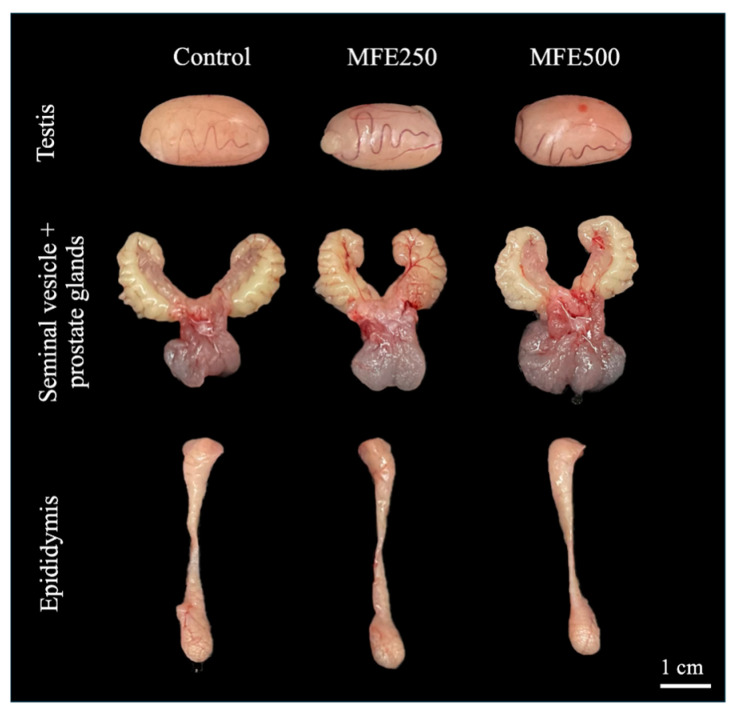
Representation of gross morphological features of reproductive organs, including testis, seminal vesicle, and epididymis, from control and MFE-treated rats for 56 consecutive days.

As shown in Figure 2, the representative testis, seminal vesicle plus prostate gland, and epididymis of the control and MFE-treated groups showed no obvious differences in gross morphological changes. It was noted that the size of the prostate gland in MFE-treated rats seemed to be slightly greater than that of the control animals (Figure 2).

### 3.4. Serum Testosterone and Seminal Vesicle Fructosamine Levels

**Figure 3:**
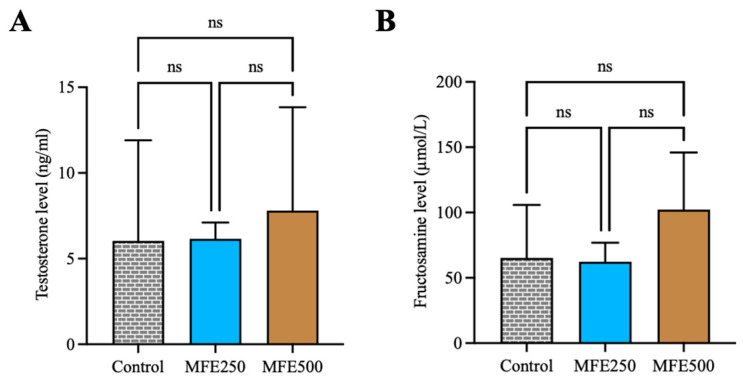
Effects of 56-Day MFE treatment (250 and 500 mg/kg) on testosterone levels in blood serum (**A**) and the fructosamine levels determined in the seminal vesicle fluid (**B**) in male rats. ns: not statistically significant.

It was found that the serum testosterone and seminal fructosamine levels tended to be increased in the MFE500 group as compared to those of the control and MFE250 groups; however, no statistically significant difference was observed (*p* > 0.05), as shown in Figure 3.

### 3.5. Effect of MFE on Seminiferous Stages VII–IX

**Figure 4:**
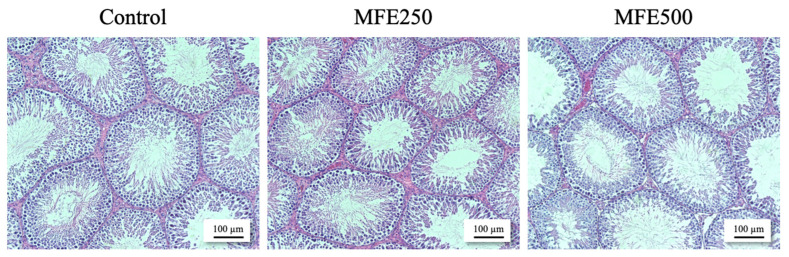
Representative histological sections of rat testes from the control and MFE-treated groups after 56 days of treatment.

As illustrated in Figure 4, the administration of MFE at doses of 250 or 500 mg/kg did not induce any histological alterations in the testicular parenchyma, including the seminiferous tubules and interstitial tissue, compared with the control group.

**Figure 5:**
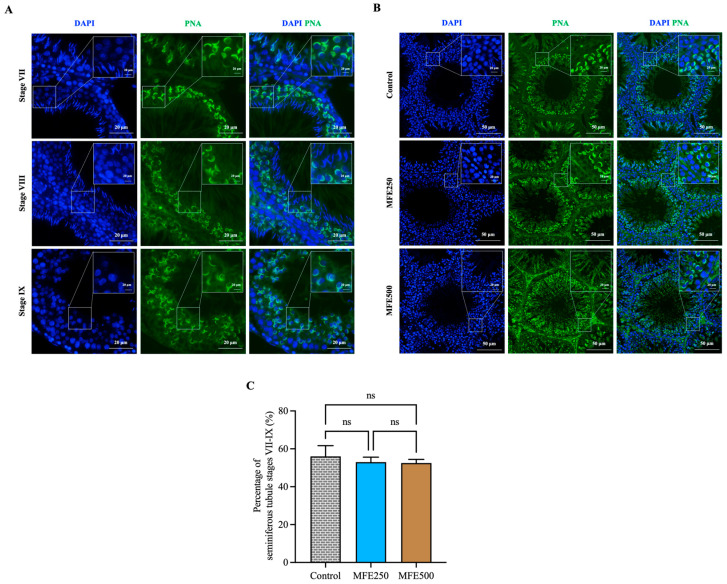
Fluorescence micrographs of PNA-lectin-stained acrosomes in developing spermatids at seminiferous stages VII–IX (**A**), representing the seminiferous tubules at stages VII–IX in each group (**B**) and quantification of the percentage of seminiferous tubules at stages VII–IX (**C**). DAPI (blue) was used as a nuclear counterstain, while PNA lectin positivity is indicated by green fluorescence. ns: not statistically significant.

Seminiferous tubule stages VII–IV, revealed by PNA lectin–acrosome staining, are shown in Figure 5A,B. The results show that the percentage of such seminiferous stages indicating spermiogenesis or sperm differentiation in spermatogenesis is not significantly different between the control and MFE-treated groups (Figure 5C).

### 3.6. Effect of MFE Treatment on Sperm Acrosome Reaction

**Figure 6:**
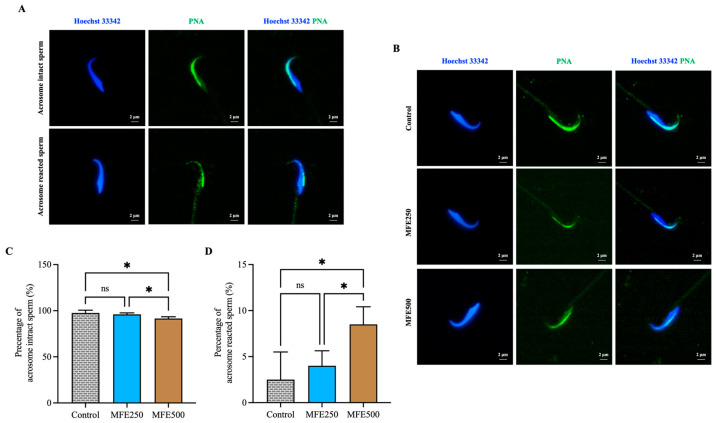
PNA lectin staining of epididymal sperm acrosomal status (**A**), representing staining sperm in each group (**B**), showing acrosome-intact (AI) and acrosome-reacted (AR) spermatozoa, and comparative analysis of the percentages of AI (**C**) and AR (**D**) among the control, MFE250, and MFE500 groups. Data are presented as mean ± S.D. * *p* < 0.05 indicates a significant difference between groups; ns: not statistically significant.

The acrosomal status of caudal epididymal spermatozoa was evaluated using PNA lectin staining (Figure 6A,B). Notably, administration of a high dose of MFE (500 mg/kg) significantly induced premature acrosome reactions in the epididymal spermatozoa, as evidenced by a reduction in acrosome-intact sperm (Figure 6C) and an increase in acrosome-reacted sperm (Figure 6D).

### 3.7. Effects of MFE on Testicular CYP11A1 and AR Expression

**Figure 7:**
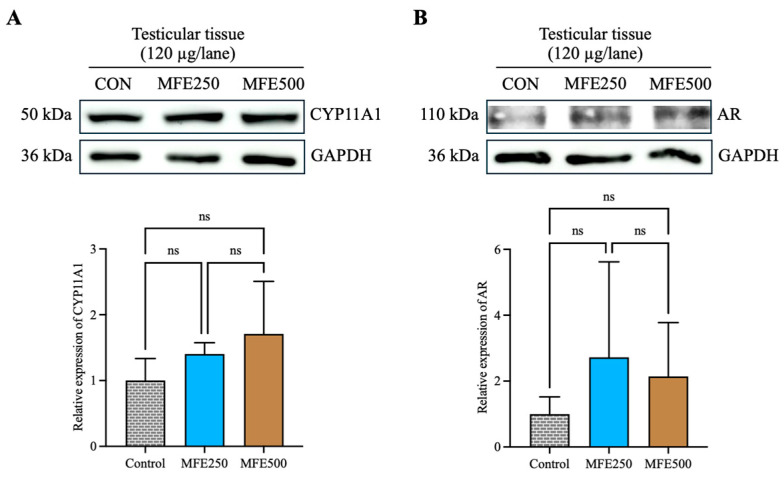
Representative testicular protein expressions with their relative intensities of CYP11A1 (**A**) and androgen receptor, AR (**B**) determined in control and MFE-treated groups. Data are represented as mean ± S.D. ns: not statistically significant.

Figure 7A shows the expressions of testicular CYP11A1 and androgen receptor proteins. As compared to the control (Figure 7A,B), the relative intensity of such protein expression tended to be increased in the MFE250 and 500 groups, but no statistically significant difference was observed (*p* > 0.05).

## 4. Discussion

This study demonstrated that Thai mulberry fruit extract (MFE) contains antioxidant capacity via DPPH and FRAP assays, along with high total phenolic, flavonoid, and anthocyanidin contents, with HPLC-confirmed cyanidin 3-glucoside (C3G) at 119.42 mg/g. These findings supported the free radical scavenging potential of plant extracts including MFE, which may contribute to protection against oxidative stress in male reproductive organs [18,42]. In the present study, the total phenolic, flavonoid, and cyanidin 3-glucoside contents quantified in the MFE were comparatively higher than values reported in a previous study, which may be attributable to differences in extraction methodology—specifically, the use of a heating process rather than freeze-drying [43]. Since polyphenols and C3G basically neutralize ROS via the donation of valence electrons to free radicals [44], they have been implicated in spermatogenic impairment [45,46]. Additionally, C3G is critical for protecting sperm DNA and membrane integrity from oxidative damage [45,47]. The doses used in this study (250 and 500 mg/kg BW) are appropriate for the evaluation of reproductive-related parameters within a subchronic toxicity study screening, per the Organisation for Economic Co-operation and Development (OECD) Test Guideline 408. Such doses have aligned with common limits for plant extracts (up to 1000 mg/kg) in safety assessments. The OECD recommends dose selection based on prior acute/subacute data to identify no-observed-adverse-effect level (NOAEL), justified by the safety profile of MFE and prior polyphenol studies [48,49].

Previous studies revealed the toxicity of phytoestrogens for the male reproductive system, resulting in reductions in blood testosterone levels and sperm quality [11,12,13,15,50]. In the present study, no significant decrease in the reproductive organ weights, sperm quality, and serum testosterone levels was observed between the control and MFE-treated groups, suggesting the non-reproductive toxicity in a cycle of rat spermatogenesis. Even though the BCF was significantly decreased in MFE250, the BCF parameter in all groups remained within the normal range for rats [51,52,53]. Along with the other CASA parameters, it is possible that treating MFE at doses of 250 and 500 mg/kg for 56 consecutive days did not affect the sperm motility patterns. The previous study demonstrated that the dried white mulberry extract could enhance sperm concentration [18]. This study also showed that the sperm concentration was significantly increased in the MFE high-dose group.

Previous studies demonstrated that exogenous estrogens could inhibit testosterone synthesis in Leydig cells, reactivate the negative feedback inhibition on testosterone production in the hypothalamic–pituitary–testicular (HPT) axis, and decrease the expression of testicular steroidogenesis proteins [9,10,54,55]. In this study, the serum testosterone levels and CYP11A1 expression did not differ between the control and MFE-treated groups, indicating that the bioactive substances of MFE did not affect the HPT axis. Moreover, the tendency for increased testosterone, CYP11A1 expression, and its functional marker (seminal fructosamine) suggests that the MFE possibly increases testosterone and seminal fructose in higher doses exceeding 500 mg/kg. Although the seminal vesicle tissue could be damaged by several substances with significantly reduced fructosamine secretion, this impairment was improved by plant extract possessing antioxidant capacity and bioactive compounds [37,56,57,58]. The absence of testicular histopathology in the control and MFE-treated groups indicated no structural damage or inflammation such as vacuolization, germ cell sloughing, or atrophy of the seminiferous tubules across the 56 consecutive exposure days. This supports the conclusion that bioactive substances in MFE could preserve the germinal architecture after treatment for 56 consecutive days, similar to those of Thai antioxidant plants, as previously demonstrated [25,59,60,61].

The lack of significant difference in the percentage of seminiferous stages (VII–IX) (spermiogenesis phases via PNA-staining) among groups indicated that MFE might not disrupt the kinetics of the spermatogenic cycle. This is because these stages are specifically where spermiogenesis (sperm differentiation) occurs [62,63]. In addition, the deprivation of testosterone caused degeneration and apoptosis in seminiferous stages VII and VIII, as previously reported, due to the toxicity of phytoestrogens [12,13,64,65,66]. The lack of histopathology at seminiferous stages VII–IX and lack of differences in the expression of AR indicated no toxicity of MFE. It was possible that the amount of phytoestrogen in MFE was not enough to disrupt testosterone synthesis [9,10,54,55]. Our results imply preserved spermatogenic cycle progression, linking to intact sperm concentration/motility, no histopathology, and a lack of germ cell loss, supporting non-interference with Sertoli–spermatid interactions. Moreover, non-significant expressions of CYP11A1 and androgen receptor (AR) in the MFE250/500 groups suggested the subtle enhancement of testosterone biosynthesis and signaling, potentially tied to trends in testosterone/fructosamine, epididymal weight gain, and sperm concentration, indicative of adaptive androgenic support without statistical power for significance [67]. Although the MFE did not significantly increase CYP11A1 and AR expression across the 56 days of treatment, increased trends were observed. It is possible that MFE at doses over 500 mg/kg may increase the expression of these proteins, along with testosterone levels. Hypothetically, this event may be directly linked to the increased epididymal weight and sperm concentration, as these parameters are highly sensitive to androgenic signaling.

The precocious acrosome reaction was observed via PNA lectin staining for the first time at high doses (MFE500). It is possible that some bioactive compounds in MFE can prematurely trigger calcium influx or cAMP signaling pathways, inducing capacitation-like changes while the sperm are stored in the cauda epididymis [46]. A previous study reported that Aspalathus linearis extract could increase spontaneous acrosome reaction, while preserving or improving other sperm quality [68]. Contrary to previous studies [69,70,71], both MFE and Aspalathus linearis, which has flavonoids, can trigger precocious acrosome reactions. It is assumed that the high dose of MFE may alter some androgen-dependent proteins that normally act as decapacitation factors to prevent premature cross-acrosome exocytosis. However, more functional assays of capacitation status, membrane integrity, or fertilization capacity should be performed to confirm this hypothesis. Consequently, while MFE increases sperm quantity, this premature loss of acrosomal enzymes may represent functional impairment that renders the sperm unable to bind and penetrate the zona pellucida [72,73,74].

## 5. Conclusions

In conclusion, Thai MFE, at doses up to 500 mg/kg, is not reproductively toxic in terms of male reproductive organ structure or sperm physiology. However, a high dose (500 mg/kg) could induce precocious acrosome reactions and might impair the sperm’s ability to fertilize an egg upon reaching the female reproductive tract. However, MFE showed potential pro-fertility effects by increasing sperm concentration and supporting androgenic markers; more functional assays such as capacitation status, membrane integrity, or fertilization capacity should be evaluated to confirm the safety of MFE use in male reproduction.

## Figures and Tables

**Table 1:** DPPH and FRAP antioxidant assays of Thai mulberry fruit extract (MFE) in comparison with antioxidant standard controls.

Samples	Antioxidant Capacity	
	DPPH: IC_50_ (mg/mL)	FRAP Value (µmol of Fe (II)/g Sample)
MFE	0.101 ± 0.002	465.01 ± 8.04
Ascorbic acid	0.004 ± 0.000	13,489.72 ± 78.91
α-Tocopherol	0.014 ± 0.000	3802.485 ± 102.14
BHT	0.095 ± 0.001	2476.44 ± 75.91

**Table 2:** Effect of 56-Day MFE treatments (250 and 500 mg/kg) on body and reproductive organ weights in male rats.

Parameters		Groups		
		Control	MFE250	MFE500
Final body weight (g)		580.6 ± 59.38	569.2 ± 47.15	553.7 ± 48.87
Testis	Absolute weight (g)	2.08 ± 0.15	2.07 ± 0.12	2.09 ± 0.10
	Relative weight (g/100 gBW)	0.36 ± 0.03	0.37 ± 0.04	0.38 ± 0.03
Epididymis	Absolute weight (g)	0.76 ± 0.06	0.78 ± 0.03	0.79 ± 0.06
	Relative weight (g/100 gBW)	0.13 ± 0.01	0.14 ± 0.01	0.14 ± 0.01 *
Seminal vesicle	Absolute weight (g)	1.36 ± 0.19	1.40 ± 0.11	1.41 ± 0.22
	Relative weight (g/100 gBW)	0.24 ± 0.05	0.25 ± 0.03	0.27 ± 0.02
	SVF secretion (%)	55.07 ± 13.26	59.66 ± 6.69	63.25 ± 3.50
Prostate glands	Absolute weight (g)	1.59 ± 0.26	1.44 ± 0.21	1.66 ± 0.28
	Relative weight (g/100 gBW)	0.28 ± 0.04	0.26 ± 0.04	0.30 ± 0.04

Data are expressed as the mean ± S.D., * *p* < 0.05 compared with the control group.

**Table 3:** Effect of 56-Day MFE treatments (250 and 500 mg/kg) on sperm quality assessed by CASA in male rats.

Sperm Parameters	Groups		
	Control	MFE250	MFE500
Motility (%)	61.44 ± 18.00	65.90 ± 15.67	62.04 ± 16.55
Progressive motility (%)	14.09 ± 5.05	15.23 ± 6.07	14.95 ± 5.08
Concentration (million/mL)	32.50 ± 17.17	36.21 ± 17.55	38.71 ± 15.04 *
ALH (µm)	18.48 ± 1.63	18.75 ± 1.65	18.71 ± 1.59
BFC (Hz)	31.43 ± 4.59	29.19 ± 3.91 *	30.84 ± 3.91 **^#^**
LIN (%)	26.94 ± 2.05	27.51 ± 2.51	27.50 ± 2.13
STR (%)	59.61 ± 5.24	60.91 ± 6.28	60.59 ± 4.70
VAP (µm/s)	133.28 ± 16.90	136.42 ± 18.63	137.63 ± 19.03
VCL (µm/s)	300.97 ± 45.05	307.14 ± 39.10	310.76 ± 46.06
VSL (µm/s)	82.89 ± 11.93	84.98 ± 12.39	86.37 ± 11.30

Data are expressed as the mean ± S.D., * *p* < 0.05 compared with the control group. **^#^** *p* < 0.05 compared with the MFE250 group. ALH; Amplitude of lateral head displacement, BFC; Beat cross frequency, LIN; Linearity, STR; Straightness, VAP; Average path velocity, VCL; Curvilinear velocity, VSL; Straight line velocity.

## Data Availability

The data are not publicly accessible due to an ongoing study. However, the data used in this study can be obtained upon request from the corresponding author.
